# Organelle inheritance and genome architecture variation in isogamous brown algae

**DOI:** 10.1038/s41598-020-58817-7

**Published:** 2020-02-06

**Authors:** Ji Won Choi, Louis Graf, Akira F. Peters, J. Mark Cock, Koki Nishitsuji, Asuka Arimoto, Eiichi Shoguchi, Chikako Nagasato, Chang Geun Choi, Hwan Su Yoon

**Affiliations:** 1 Department of Biological Sciences, Sungkyunkwan University, Suwon, 16419 Korea; 2 Bezhin Rosko, 29250 Santec, France; 3 Algal Genetics Group, UMR 8227, CNRS, Sorbonne Universités, UPMC, Station Biologique Roscoff, CS 90074, 29688 Roscoff, France; 4 Marine Genomics Unit, Okinawa Institute of Science and Technology Graduate University, Onna, Okinawa 904-0495 Japan; 5 Muroran Marine Station, Field Science Center for Northern Biosphere, Hokkaido University Muroran, 051-0013 Muroran, Hokkaido, Japan; 6 Department of Ecological Engineering, Pukyong National University, Busan, 48513 Korea; 7 Present Address: Marine Biological Laboratory, Graduate School of Integrated Sciences for Life, Hiroshima University, Onomichi, Hiroshima 722-0073 Japan

**Keywords:** Evolutionary theory, Molecular evolution, Phylogenetics

## Abstract

Among the brown algal lineages, Ectocarpales species have isogamous fertilization in which male and female gametes are morphologically similar. In contrast, female gametes are much larger than male gametes in the oogamous species found in many other brown algal lineages. It has been reported that the plastids of isogamous species are biparentally inherited whereas the plastids of oogamous species are maternally inherited. In contrast, in both isogamous and oogamous species, the mitochondria are usually inherited maternally. To investigate whether there is any relationship between the modes of inheritance and organellar genome architecture, we sequenced six plastid genomes (ptDNA) and two mitochondrial genomes (mtDNA) of isogamous species from the Ectocarpales and compared them with previously sequenced organellar genomes. We found that the biparentally inherited ptDNAs of isogamous species presented distinctive structural rearrangements whereas maternally inherited ptDNAs of oogamous species showed no rearrangements. Our analysis permits the hypothesis that structural rearrangements in ptDNAs may be a consequence of the mode of inheritance.

## Introduction

The brown algae (Phaeophyceae) are a group of photosynthetic heterokonts (=stramenopiles), a secondary endosymbiotic lineage containing red algal-derived plastids^1^. The four brown algal orders analyzed in this study, Ectocarpales, Fucales, Laminariales, and Dictyotales, have macroscopic thalli and are distributed worldwide in low-to-mid latitudes where they play important roles in marine ecosystems. Also, they have great potential for commercial uses such as food or in other seaweed industries. From a taxonomic point of view, the orders Ectocarpales, Fucales and Laminariales are grouped into the subclass Fucophycidae, which is defined as a lineage derived from the brown algal crown radiation (BACR), whereas Dictyotales is classified into the Dictyotophycidae^2^ (Fig. S1).

The life cycles of most brown algae, including species of the Laminariales and Ectocarpales, are characterized by an alternation between a diploid sporophyte and a haploid gametophyte (i.e., a diploid-haploid life cycle). In contrast, in the Fucales meiosis occurs in the parental diploid plants and they directly produce gametes (i.e., a diplontic life cycle)^3,4^. Three fertilization types can be defined based on the morphologies and flagella of gametes, independent of whether they are produced via a gametophyte stage or not: the isogamous type in which flagellated gametes are morphologically similar; the anisogamous type where one flagellated gamete is larger than the other; and the oogamous type in which one gamete is a non-motile cell called the egg cell (or oocyte) and the other is a smaller, flagellated sperm cell^5^. Most brown algae, including the Laminariales and most species of the Fucales, are oogamous. Anisogamy is only observed in a few lineages such as the Onslowiales, Asterocladales, Nemodermatales, and in some Fucales, Sphacelariales and Ectocarpales species. With the exception of the orders Ectocarpales and Sphacelariales, isogamy is observed only in some species-poor orders such as the Ralfsiales, Scytothamnales, Ascoseirales, Syringodermatales and Ishigeales. Most of the 750 Ectocarpales species are considered to be isogamous^1,6^, although some of these species exhibit a very low level of anisogamy, for example the slightly larger female gametes of *Ectocarpus* species^7^ and near-isogamy in *Scytosiphon* species^8^. However, oogamy has not been reported in the Ectocarpales. Remarkably, all three types of fertilization have been observed in the Sphacelariales, but asexual reproduction is also common in this order^9^.

During sexual reproduction, cellular organelles (i.e., mitochondria and plastids) are transmitted to the next generation following several different modes of inheritance. Uniparental inheritance (especially maternal inheritance) of organelles is observed in the majority of brown algae^10,11^. Ultrastructural studies for the brown algae *Laminaria angustata* Kjellman, *Fucus vesiculosus* Linnaeus, and *F. distichus* Linnaeus demonstrated uniparental inheritance of the organelles^12–14^. Furthermore, analysis of cross-species hybrids confirmed that only mitochondria and plastids from the female gamete are transferred to the zygote during fertilization for both the Laminariales and the Fucales^15,16^. These results indicate that organelles are maternally inherited in the oogamous brown algae.

The situation is more complex in isogamous species. Biparental plastid inheritance has been reported in several Ectocarpales species^17–19^. In isogamous Ectocarpales species, mitochondria are commonly maternally inherited (demonstrated for *Ectocarpus siliculosus* and *Scytosiphon lomentaria* Lyngbye^20–22^) but unusual patterns of mitochondrial inheritance such as paternal inheritance or random paternal or maternal inheritance have been described for some strains of *Ectocarpus*^23^.

Taken together, these observations suggest that the type of fertilization (i.e., isogamy or oogamy) may influence the mode of organelle inheritance. This interesting question was posed in a previous study^24^. However, Crosby and Smith only compared genome size and gene compactness and did not take into consideration the physiological properties of species. Biparental inheritance of plastids appears to be associated with isogamy whereas oogamous species exhibit maternal inheritance of this organelle, at least in the brown algal lineages that have been studied. Accordingly, Ectocarpales species are expected to have unique properties because they exhibit isogamous gametes and biparental organelle inheritance.

Despite these interesting features of the Ectocarpales, only two plastid genomes (ptDNAs) are currently available in the NCBI database from this order (*E. siliculosus*, *Pleurocladia lacustris* A.Braun), whereas ptDNAs from three Laminariales species (*Costaria costata* (C. Agardh) De A. Saunders*, Saccharina japonica* (Areschoug) C.E. Lane, C. Mayes, Druehl & G.W. Saunders and *Undaria pinnatifida* (Harvey) Suringar) and four Fucales species (*Coccophora langsdorfii* (Turner) Greville, *F. vesiculosus, Sargassum horneri* (Turner) C. Agardh and *Sargassum thunbergii* (Mertens ex Roth) Kuntze) are available. Furthermore, the two Ectocarpales species belong to the same family, the Ectocarpaceae, whereas the three Laminariales species are classified into three distinct families and the four Fucales species represent two families. Therefore, more ptDNA sequence data are needed for the Ectocarpales species to study the evolution of ptDNA in relation to inheritance mode.

We characterized six complete ptDNAs from five Ectocarpales species: *Scytosiphon promiscuus* McDevit & G.W. Saunders (from both male and female gametophytes), *Sc. canaliculatus* (Setchell & N.L. Gardner) Kogame, *Petalonia binghamiae* J. Agardh, *Cladosiphon okamuranus* Tokida and a male gametophyte strain of *E. siliculosus* (see the taxonomic classification described in previous reports^2,25,26^). The ptDNA sequence of the Dictyotales species *Dictyopteris divaricata* (Okamura) was used as an outgroup. Using a total of 16 ptDNAs including 10 available ptDNAs, we compared genomic variations at the order-, family-, genus- and species-level to understand rearrangement events and the evolutionary history of organelle genomes.

## Results and Discussion

### General features of brown algal plastid genomes

Six ptDNAs from *Cl. okamuranus* (137,324 bp), *Sc. canaliculatus* (133,508 bp)*, Sc. promiscuus* (female: 134,358 bp, male: 134,366 bp), *Pe. binghamiae* (136,274 bp) and *E. siliculosus* (139,516 bp) were completely sequenced in this study. Information regarding the sequencing results and assemblies of the six ptDNAs is summarized in Table S1 and the circularized ptDNA maps are shown in Fig. S2. The average ptDNA GC content ranged from 30.2% (*Cl. okamuranus*) to 31.3% (*Sc. promiscuus*). The ptDNA of *Cl. okamuranus* contained 145 CDSs including two conserved hypothetical ORFs (which are also found in sister species but not homologous with any records in the NCBI database), and six rRNA and 29 tRNA genes. The *Sc. canaliculatus* and *Sc. promiscuus* ptDNAs both contained 143 CDSs including three conserved hypothetical ORFs and six rRNA, however the *Sc. promiscuus* ptDNA (28 tRNAs) had one more tRNA than that of *Sc. canaliculatus* (27 tRNAs). Finally, the *Pe. binghamiae* ptDNA contained 143 CDSs including two conserved hypothetical ORFs, six rRNA, and 28 tRNA genes. There were some differences in both size and content between the ptDNAs of the *Ectocarpus* species-7 strain Ec32 (NC_013498.1; note that Ec32 was originally defined as *Ectocarpus siliculosus* but has recently been shown to correspond to another species, designated *Ectocarpus* species-7^26^) and the *E. siliculosus* strain Ec08 (this study). For instance, the inverted repeat (IR) region of the ptDNA of *E. siliculosus* had an additional tRNA gene compared with that of *Ectocarpus* species-7.

Table 1 compares the six ptDNAs described above and ten additional ptDNAs from the NCBI database (https://www.ncbi.nlm.nih.gov/; see also Fig. S2 for the presence and absence of individual genes). The numbers of CDSs (139–143) and tRNAs (27–32) showed relatively high variation within the Ectocarpales species compared to the highly conserved gene contents of the other brown algal lineages (CDSs: 137, tRNAs: 28 or 29 in four Fucales, three Laminariales and one Dictyotales species, see Table 1). The transfer-messenger RNA (tmRNA), which forms a ribonucleoprotein complex together with small protein B (*smp*B), elongation factor Tu (EF-Tu), and ribosomal protein S1, was found in all Ectocarpales species, whereas this gene was only found in *Sar. horneri* among the Fucales and absent in the Laminariales. In total, the six Ectocarpales ptDNAs shared 15 hypothetical CDSs (i.e., *ycf* genes). Of these, *ycf*17 and *ycf*54 gene were missing in the Fucales and Laminariales, respectively, so that the latter only had 14 hypothetical CDSs. In addition, the *syf*B gene was missing in the Fucales and the *pet*L gene was absent in the Laminariales species (Table S2).

**Table 1:** The genomic contents and general features of twelve brown algal ptDNAs.

	Ectocarpales								Laminariales	Fucales	Dictyotales
Species	***Cladosiphon okamuranus***	***Scytosiphon canaliculatus***	***Scytosiphon promiscuous*** **(Male)**	***Scytosiphon promiscuus*** **(Female)**	***Petalonia binghamiae***	***Ectocarpus siliculosus*** **(Ec08, Male)**	*Ectocarpus* species-7 (Ec32, Female)	*Pleurocladia lacustris*	*Undaria pinnatifida* *Costaria costata* *Saccharina japonica*	*Fucus vesiculosus* *Coccophora langsdorfii* *Sargassum thunbergii* *Sargassum horneri*	*Dictyopteris divaricata*
NCBI Accession	**—**	**—**	**—**	**—**	**—**	**—**	NC_013498.1	NC_032045	NC_028503.1 NC_028502.1 NC_018523.1	NC_016735.1 NC_032288.1 NC_029134.1 NC_029856.1	NC_036804.1
Genome size(bp)	**137,324**	**133,508**	**134,366**	**134,358**	**136,274**	**139,516**	139,954	138,815	129,947–130,584	124,068–124,986	126,099
GC %	**30.2**	**31.1**	**31.3**	**31.3**	**31.2**	**30.8**	30.7	29.8	30.6–31.1	28.9–30.6	31.2
CDSs*	**143**	**140**	**140**	**140**	**141**	**142**	142	139	137	137	137
rRNA	**6**	**6**	**6**	**6**	**6**	**6**	6	6	6	6	6
tRNA	**29**	**27**	**28**	**28**	**28**	**32**	32	30	28–29	28	28
tmRNA	**1**	**1**	**1**	**1**	**1**	**1**	1	1	0	0–1	1
**IR region features**											
Length	**15,803**	**9,799**	**11,258**	**11,257**	**14,731**	**17,346**	17,220	16,168	10,030–10,818	10,740–10,892	11,990
GC%	**38.4**	**44.7**	**42.9**	**42.9**	**31.2**	**39.5**	38.7	39.6	43.7–45.3	43.0–43.4	40.7
CDSs (partial)	**8**	**2**	**2**	**2**	**4**	**6**	6	2 (2)	0 (4)	0 (4)	2 (4)
tRNA	**4**	**3**	**4**	**4**	**4**	**10**	8	8	4	4	4

In total, 16 available ptDNAs were compared. The general features of newly characterized species are represented in bold. The IR region values include features of two IR regions.

All brown algal ptDNAs share a similar architecture, with two IR regions separated by a small single copy (SSC) and a large single copy (LSC) region (see Fig. S2). Each IR region contains one ribosomal RNA operon consisting of 16S, 23S and 5S rRNA and two tRNAs between the 16S and 23S rRNA. Interestingly, noticeable genome size and gene number variation in the Ectocarpales ptDNAs were positively correlated with the length of the IR regions and with the number of genes within these regions (Table 1). For instance, *Sc. canaliculatus*, which contained the smallest IR region (9,799 bp), had the smallest ptDNA (133,508 bp), whereas due to the high number of tRNAs in the IR region (8–10 tRNAs), *E. siliculosus* was the most tRNA-rich species among the Ectocarpales taxa studied (Table 1). However, variations between IR regions did not explain all the differences between the Ectocarpales ptDNAs: *Ectocarpus* species and *Pl. lacustris* had the same number of tRNAs in their IR regions, but *Ectocarpus* species contained two more tRNAs in total, located in the LSC region. Overall, isogamous Ectocarpales species presented a much higher level of ptDNA variation than the oogamous brown algal species. Furthermore, the IR and its neighboring regions appeared to be a hot spot for variations in both genome size and gene number.

### Plastid genome expansion in brown algae

**Figure 1:**
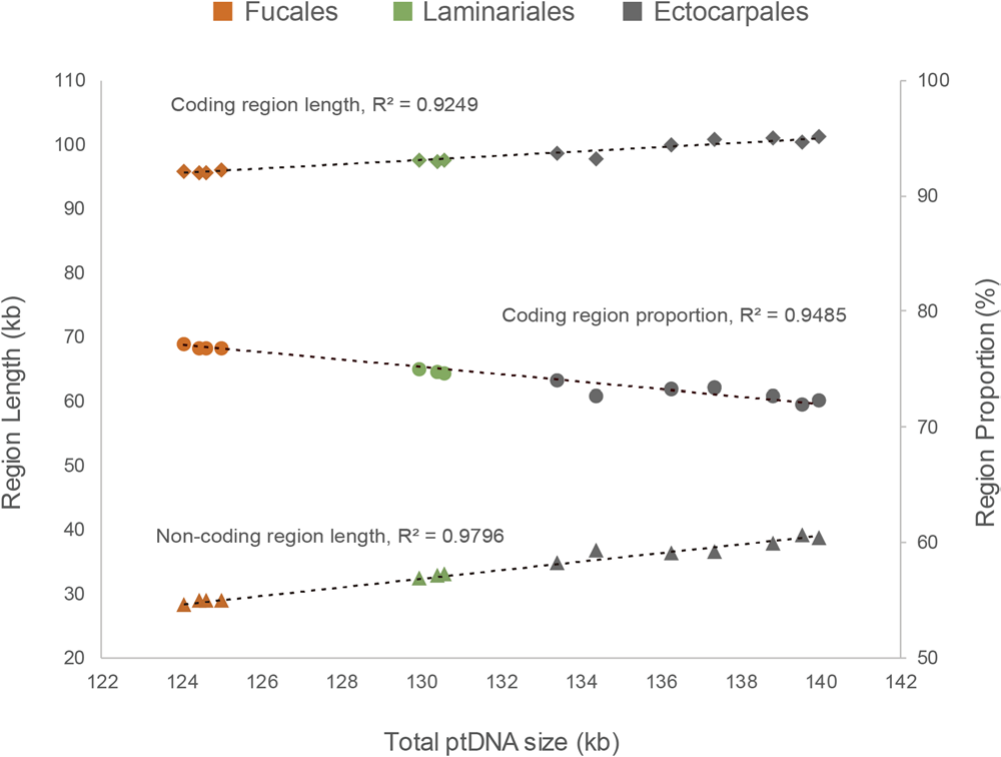
Total length of non-coding DNA sequences regressed on plastid genome size. There was a strong correlation (*R*^2^ = 0.9796) between the amount of non-coding DNA sequence and whole plastid genome size for the 12 brown algae. The dashed line indicates the linear regression representing the correlation. Data points for the Fucales (brown) and Laminariales (green) species are positioned closely together but data points for the Ectocarpales species (grey dots) are widely scattered.

In an effort to identify factors that might explain the variability in ptDNAs of Ectocarpales species, we plotted length of non-coding sequences with genome size for each ptDNA (Fig. 1). *D. divaricata* was excluded from this analysis because this species was the only member of the order Dictyotales analyzed (see Fig. S1). The Fucales and Laminariales species showed conservation of both ptDNA size (Fucales: 124,068–124,986 bp; Laminariales: 129,947–130,584 bp) and the length of the non-coding regions (Fucales: 28,318–28,975 bp, Laminariales: 32,484–33,070 bp), forming two clusters in Fig. 1. In contrast, the Ectocarpales species exhibited wide variations in ptDNA size (133,508–139,954 bp) as well as in the amount of non-coding sequence (34,104–39,737 bp). Nevertheless, we cannot exclude the possibility that other taxa have conserved ptDNA sizes and there has been less loss of non-coding DNA from the Ectocarpales ptDNAs.

A previous molecular clock analysis^6^ estimated that the extant Ectocarpales species diverged 70 MYA (million years ago). Fucales species have been diverging for a similar period of time (65 MYA) but have markedly more conserved ptDNAs than the Ectocarpales (note that Laminariales species diverged more recently, about 30 MYA). This difference could be due to either a higher level of conservation in the Fucales, or a higher level of variation in the Ectocarpales. In any event, this analysis demonstrated that the isogamous Ectocarpales species have higher size variation in ptDNA (about maximum 10-fold range) than do the oogamous Fucales and Laminariales species.

A strong positive correlation was observed between ptDNA size and the total length of non-coding sequence (*R*^2^ = 0.9796, see Fig. 1). In addition, at least within the subclass Fucophycidae, ptDNA size variation was correlated with phylogenetic relationship. With the Dictyotales as the outgroup, the Laminariales and Ectocarpales were more closely related to each other than to the Fucales in the phylogeny (Fig. S1). Interestingly, the Fucales ptDNAs were the smallest, the Laminariales ptDNAs of medium size and the Ectocarpales ptDNAs were the largest, suggesting an unique increase in non-coding sequence content during evolution from the ancestor of the Ectocarpales.

Comparative analysis of ptDNAs from a broad range of photosynthetic species has led to the suggestion that there is a relationship between the mode of plastid inheritance and plastid genome architecture^24^. However, no significant correlations have been found so far, except for paternally inherited ptDNAs from a specific land plant phylum (Pinophyta, Viridiplantae). Another study found a correlation between genome size and non-coding sequence content in mitochondrial genomes^27^, but evolutionary trends of genome size variation were not considered. Plastid genomes generally decrease in size in an irreversible manner^28^. However, according to our analysis, brown algal ptDNAs or at least those of isogamous Ectocarpales, have not followed this general trend of plastid genome evolution although it is still difficult to rule out sequence loss in the other lineages.

### Alignments of brown algal ptDNAs identify order- and family-level variation

**Figure 2:**
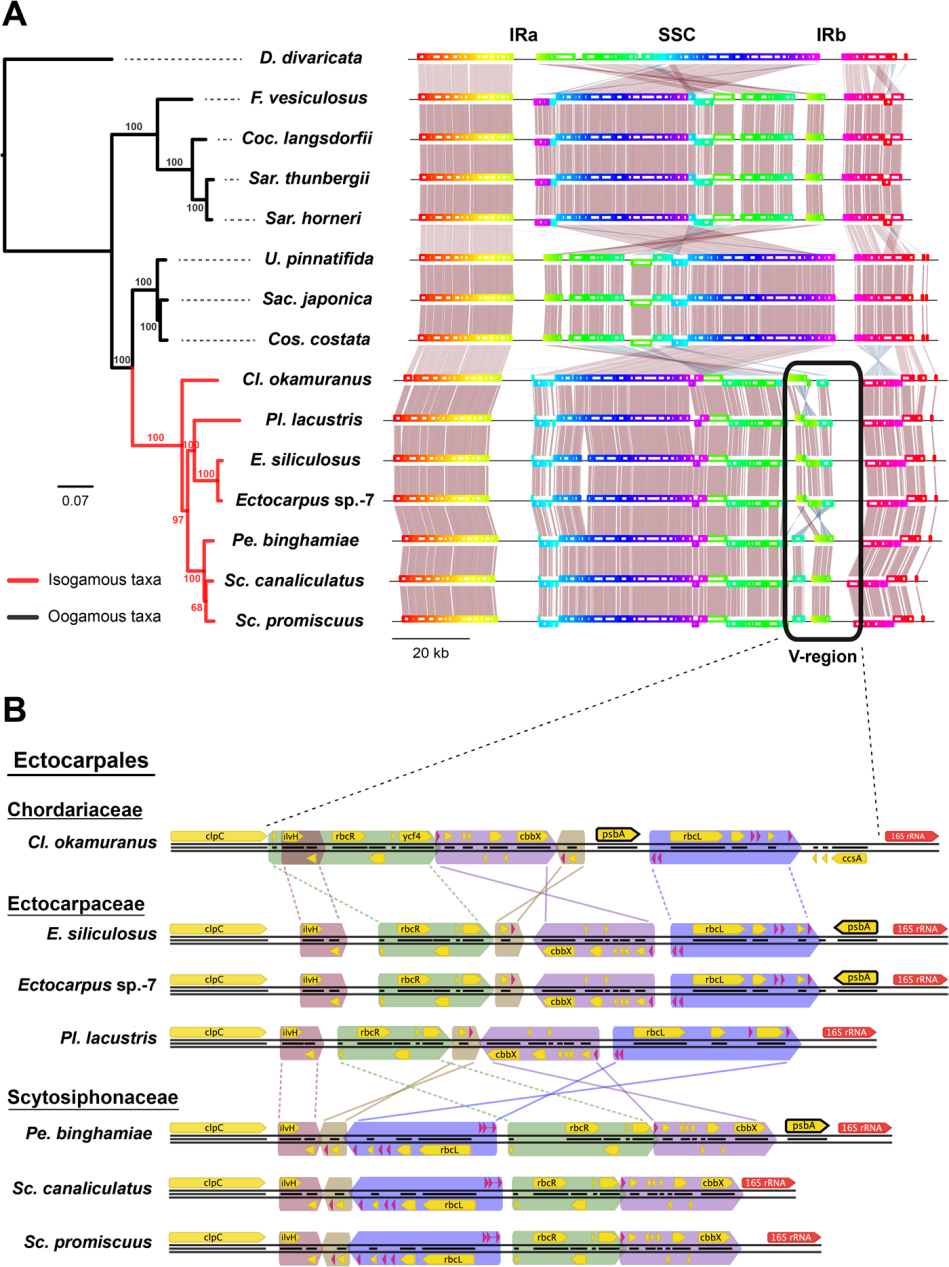
Co-linear alignment of ptDNAs. (**A**) Alignment of ptDNAs from 15 species of brown algae. The rainbow color painted along the gene annotation indicates structural changes including translocations and inversions. Only the ptDNA from the female strain is shown for *Sc. promiscuus*. The phylogenetic relationships between species are based on a maximum likelihood (ML) analysis using 137 concatenated core genes. Only the ptDNAs of the isogamous clade (Ectocarpales, red branches) show high rearrangement. The black round rectangle represents the V-region which is highly rearranged near the IRb region. (**B**) Gene synteny within the V-region (the highly variable region) in ptDNAs of Ectocarpales species. The colored arrows indicate conserved gene order and its direction. Lines drawn between alignments show translocations of syntenic blocks with inversion (solid lines), or without inversion (dashed lines).

To investigate the structural rearrangements that occurred between the 16 ptDNAs, we performed co-linear analysis using full length alignment of ptDNAs (Fig. 2A). A unique, single syntenic block (i.e., identical gene order between the species) was found for the four Fucales ptDNAs and for the three Laminariales ptDNAs. The conserved synteny in each order indicated that no rearrangements had occurred within those lineages. In contrast, the five Ectocarpales ptDNAs showed several inversions and translocations that were located within a specific variable region within the SSC (approximately 15 kb in length, hereafter called the V-region as shown in Fig. 2A).

To further investigate structural variations in the Ectocarpales ptDNAs, we focused our analysis on syntenies of the V-region. The species within the families Ectocarpaceae (*E. siliculosus, Ectocarpus* sp.-7 and *Pl. lacustris*) and Scytosiphonaceae (*Sc. canaliculatus*, *Sc. promiscuus* and *Pe. binghamiae*) shared synteny in the V-region. In contrast, the ptDNA of *Cl. okamuranus*, of the family Chordariaceae, exhibited a different organization to those from other families (Fig. 2B). ptDNAs of species from the same family had syntenic blocks that contained several genes arranged in the same order. However, between families, ptDNAs exhibited several inversions and translocations. The only exception to this intra-family conservation was for the *psb*A gene, which appears to have been present in the IR region of the common ancestor of Ectocarpaceae and Scytosiphonaceae but to have been lost independently in *Pl. lacustris* and *Scytosiphon* species. (Fig. 2B).

### Comparisons of IR region configuration identified genus-level variation

**Figure 3:**
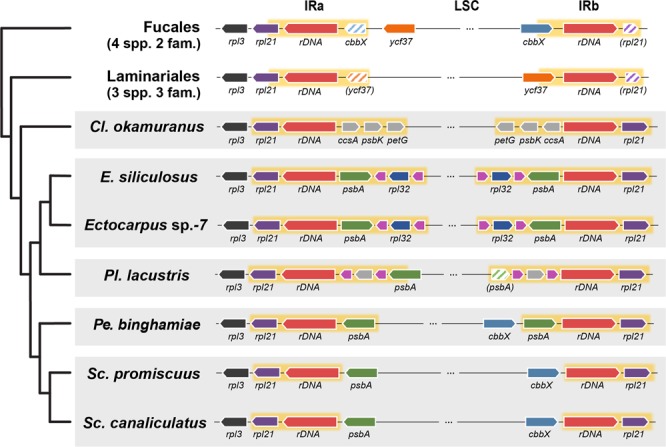
Configuration of the IRs and their neighboring regions. IRa, IRb and the neighboring region of all brown algal ptDNAs are shown. The yellow box indicates the IR region and the red block indicates two ribosomal RNA operons (including two tRNAs). Duplicated partial CDSs in the IR regions are hatched and the name is shown in parentheses. The purple blocks without a name indicate tRNAs.

We investigated the gene configuration within the IR and its neighboring regions to find additional small variations similar to the absence of the *psb*A gene (Fig. 3), using the Fucales and Laminariales species for comparison. Several partial CDS sequences were identified in the IR regions. The four ptDNAs from the Fucales and the three from the Laminariales shared the same configuration within the IR region, which contained one copy of an rRNA operon and two paired, partial CDSs of *rpl*21-*cbb*X and *rpl*21-*ycf*37 within each group, respectively. In contrast, each of the five Ectocarpales genera exhibited a unique IR region configuration. For instance, the *Cl. okamuranus* IR region included four complete CDSs, but *Scytosiphon* species had only one. Because rRNA operon regions have higher GC content than protein-coding regions, *Scytosiphon* species have noticeably higher GC content in the IR region (above 40%, see Table 1 for comparison). Among the Ectocarpales, only the *Pl. lacustris* IR region had a partial CDS (i.e., *psb*A) and the *Pl. lacustris* and two *Ectocarpus* species IR regions had more tRNA encoding genes than in other species.

Inverted repeat sequences are known to play an important role in plastid genome rearrangement events^29–31^. Variation at the IR boundary, including rearrangement of gene order and duplication of genes, are usually explained by double-strand breaks and repair involving repeated sequences. Partial CDSs are usually found in the IR regions because of the duplicated sequences at IR boundaries^32,33^. The species-specific differences in ptDNAs between Ectocarpales species suggest the occurrence of rearrangement within or around the IR regions. Moreover, it is noteworthy that the IR regions were only highly variable in the Ectocarpales whereas the IR regions of the Laminariales and the Fucales appear to be stable, suggesting that frequent ptDNA rearrangement events are associated with biparental plastid inheritance and isogamy.

### Differences between the ptDNAs of two recently diverged *Ectocarpus* species; *E. siliculosus* and *Ectocarpus* species-7

**Figure 4:**
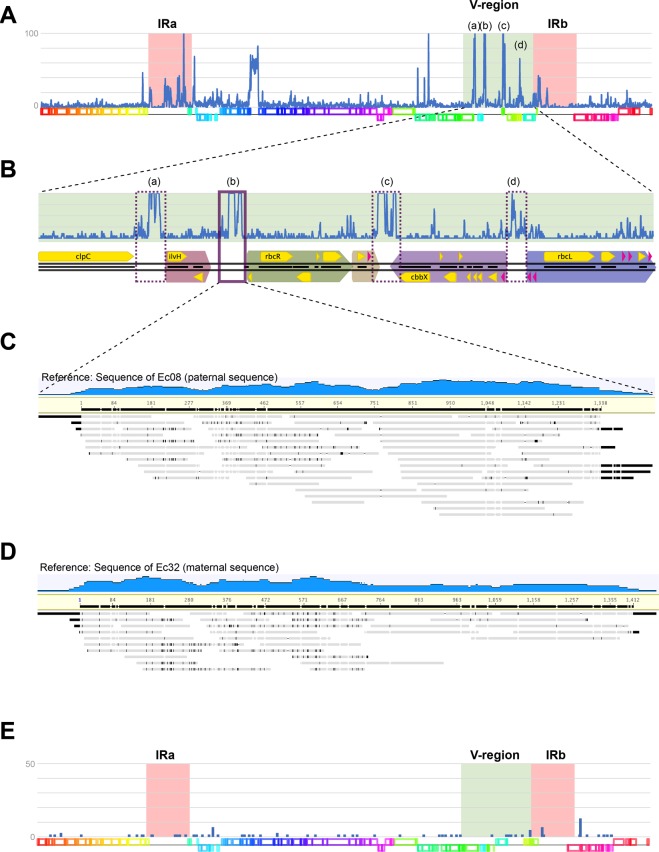
Nucleotide substitution rates per site between ptDNAs of two *Ectocarpus* species. (**A**) Nucleotide substitution frequency (counts per each window) between ptDNAs of two species of *E. siliculosus* plotted on the full ptDNA sequence. The window size is 100 bp and the maximum value is 100. There are several peaks of high variability especially in the IRs and the V-region. Four major peaks: (a) to (d) in the V-region are magnified in panel B. (**B**) Each peak corresponds to the flanking region of a syntenic block described in Fig. 3 (violet boxes). (**C**,**D**) Biparental inheritance of ptDNA was confirmed by mapping sequence reads from a hybrid onto region (b) of the parental ptDNA sequences. (**E**) Nucleotide substitution plot between male and female *Sc. promiscuus* individuals from the same population. The window size is 100 bp and the maximum value is 50. There were few substitutions between the two individuals.

We compared ptDNAs of two *Ectocarpus* species (i.e., *E. siliculosus* and *Ectocarpus* species-7) and two ptDNAs of male and female *Sc. promiscuus* strains from the same site (Hokkaido, Japan). The two *Ectocarpus* ptDNAs had very similar compositions (see Table 1) and were completely syntenic (see Fig. 2). Figure 4A shows the frequency of nucleotide substitutions between the two genomes along a pairwise alignment. There were four major peaks of nucleotide substitution (i.e., highly variable sites) in the V-region, located in intergenic regions (Fig. 4B). Not every intergenic region exhibited a high level of variation, but the intergenic region between the syntenic blocks represented in Fig. 2B was highly variable, implying these sites are hot spots of variation. This result shows species-level variation that represents an initiation of variation observed more broadly in Ectocarpales ptDNAs. To confirm the biparental inheritance of ptDNAs and to verify the presence of both maternal and paternal ptDNA in the filial individual, we mapped PGM reads generated from the genomic DNA of the hybrid (i.e., Ec08: male x Ec73: female) onto the (b) regions of the parental ptDNAs of the male and female parents (Fig. 4C,D; C: male; D: female). The even and continuous coverage of read mappings both on the maternal (i.e., *Ectocarpus* species-7) and paternal (i.e., *E. siliculosus*) ptDNAs indicates that both ptDNAs were present in the hybrid sporophytic tissues after the fertilization and that both ptDNAs are replicated during cell division and growth.

As expected for an intra-specific comparison, the ptDNAs of male and female plants of *Sc. promiscuus* from the same population showed much less variation, even in the V-region (Fig. 4E). Very few single nucleotide polymorphisms (SNPs) and indel variations were found between male and female plants especially in the intergenic regions (genic region 0.02% vs. intergenic region 0.14%).

### Alignments of brown algal mtDNAs

Two new circular mtDNA sequences of *Pe. binghamiae* (38,160 bp) and *Cl. okamuranus* (38,419 bp) were also completed in this study (Fig. S3). The gene contents of the two mtDNAs were similar (*Pe. binghamiae*: 38 CDSs, 24 tRNAs, and 3 rRNAs; *Cl. okamuranu*s: 37 CDSs, 24 tRNAs, and 3 rRNAs) and the two sequences had almost identical GC contents (*Cl. okamuranus*: 34.3%; *Pe. binghamiae*: 34.4%). Although there were long intergenic sequences in the *Pylaiella littoralis* mtDNA, an analysis of synteny with other available brown algal mtDNAs indicated strong conservation of genome structures across all taxa, regardless of the type of fertilization (isogamous Ectocarpales and oogamous Fucales, Laminariales, Dictyotales, and Desmarestiales; Fig. S4), which is consistent with previous reports^27,34,35^.

### Why are only ectocarpales ptDNAs structurally variable?

**Figure 5:**
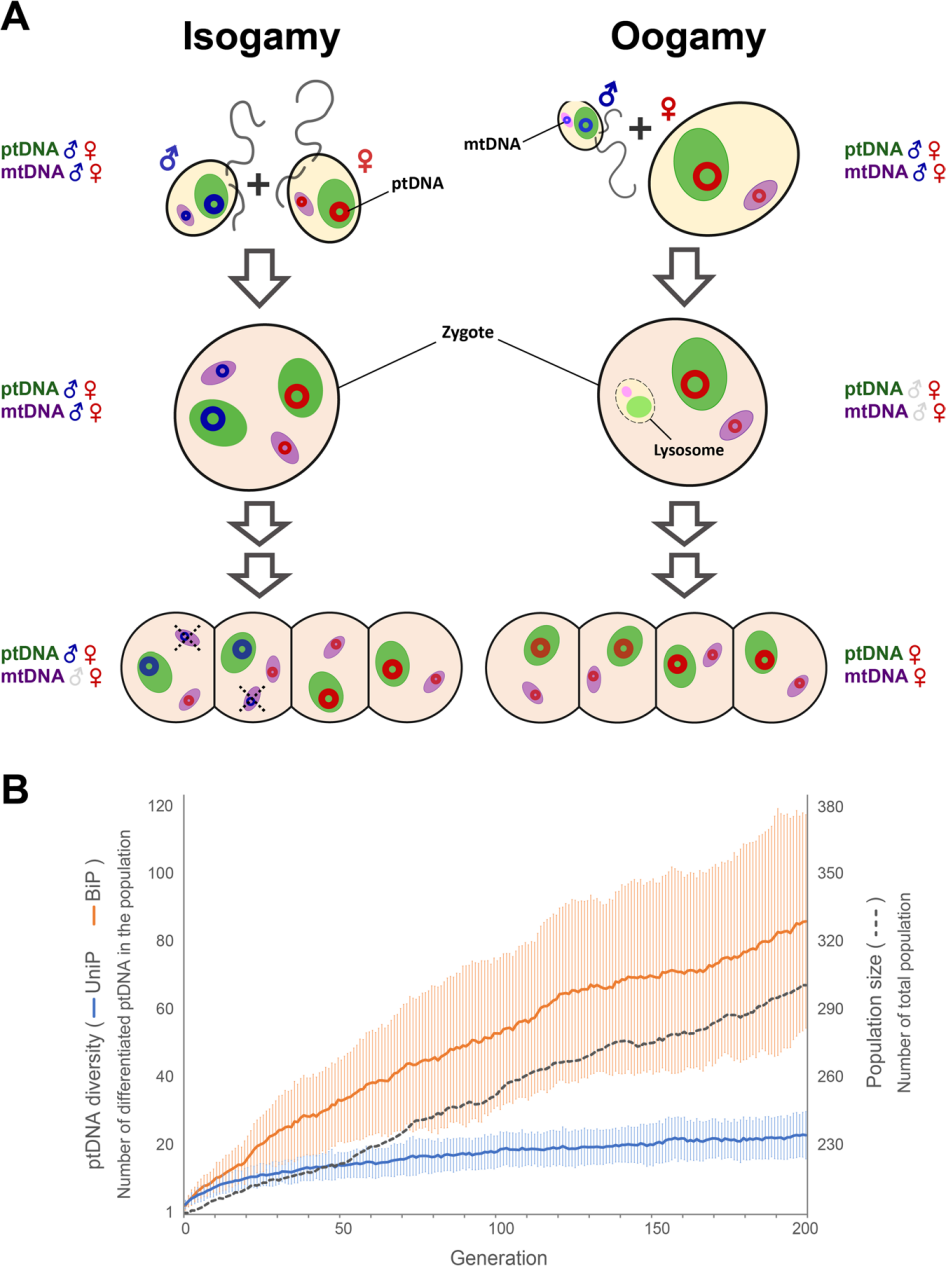
Organelle inheritance in isogamous and oogamous brown algae. (**A**) The process of organelle inheritance and elimination. Red and blue circles indicate circular DNA from female and male gametes. Mitochondria are eliminated at the zygote stage in oogamous species or at the 4-cell sporophyte stage in isogamous species. Only maternal inheritance of mitochondria is shown for isogamous species, but note that paternal inheritance has been detected in some strains of *Ectocarpus*^23^. (**B**) Simulation of changes in ptDNA diversity (number of differentiated ptDNA) in two populations under the same conditions except for the inheritance mode of plastids (biparental or uniparental). Starting with a single original ptDNA genotype, the diversity of ptDNA steadily increases due to mutations in both populations, but this occurs much more rapidly in the biparental (BiP) population. The increase in ptDNA diversity in the BiP population (Orange line) is proportional to the increase in population size, while the diversity in the uniparental (UniP, Blue line) population increases more slowly because half of the modified ptDNA is eliminated. Error bars for each population show standard deviations of 100 replicated results. The script used for simulation is in Supplementary Information with instructions.

In the brown algae, the type of fertilization between gametes seems to determine the mode of organelle inheritance. Figure 5A summarizes the process of organelle inheritance in isogamous and oogamous brown algae. Although the details of the inheritance mechanisms are different, both plastids and mitochondria of oogamous species, but only mitochondria of isogamous species are uniparentally inherited^23,36–38^. Our data indicate that ptDNAs exhibit more structural variations in isogamous Ectocarpales species than in oogamous brown algal species. Furthermore, a high level of structural variation was only observed for the biparentally-inherited ptDNAs and not for the uniparentally inherited mtDNAs of the Ectocarpales. This suggests a correlation between biparental inheritance and genomic rearrangements, implying that the mode of inheritance may affect genome structure, at least in brown algae.

Rearrangement occurred mostly around the IR regions. For example, we found more structural variation in the IR and its neighboring V-regions in the Ectocarpales. Since the variations were found at the genus and species levels, this structural variation appears to be an on-going process that is likely to include recent rearrangement events. Genomic rearrangement during replication is a common consequence of repair mechanisms^39,40^. It was reported that recombination of plastid genomes is the result of a copy-dependent repair system in an angiosperm family, the Geraniaceae^41^. Also, biparental inheritance is thought to represent an important source of heteroplasmy of organellar genomes^42,43^. These ideas also make explicit statements about causality between the mode of inheritance and the genomic variation.

A previous study of artificial hybrids between pairs of *Ectocarpus* species^21^ indicated that both parental ptDNAs are present in the zygote cell before the first cell division creating a possibility for recombination and exchange of genetic material. For recombination to occur between plastid genomes, they must come into physical contact, for example, as a result of plastid fusion. However, physical contacts between two parental plastids, including fusions, have not been observed during zygote stage, and the chimeric nature of the developing sporophytes indicates that plastids of different parental origins are segregated into different cells during the first cell division of the sporophyte. Therefore, it is not clear whether recombination can occur between ptDNAs during fertilization and development.

Based on the results of this study, we hypothesize that ptDNA variability in isogamous brown algae is a consequence of biparental plastid inheritance. When inheritance is uniparental, plastids are received from only one of the two parents whereas with biparental inheritance the progeny receive plastids from both the male and the female parent. Biparental inheritance allows the transmission of variants that have arisen in both the male and female lineages, effectively doubling the capacity of the system to transmit ptDNA variations to the next generation. To illustrate our hypothesis, we conducted a simple computational simulation of ptDNA variation and inheritance in both biparental (BiP) and uniparental (UniP) populations, respectively. The results of the simulation are presented in Fig. 5B. Those virtual populations do not exactly reflect actual populations, but they represent fairly well the effect of mode of plastid inheritance, because other factors that can affect ptDNA diversity are controlled. Total population size was gradually increased at the same rate in both populations, whereas ptDNA diversity increased much more rapidly in the BiP population compared to the UniP population, supporting our hypothesis.

## Conclusion

We hypothesize that heteroplasmic variation due to errors in genome replication or damage repair may be inherited by the next generation and accumulate gradually in the ptDNAs of brown algae over generations. In species that exhibit biparental inheritance, such as isogamous brown algae, each individual of the population can potentially transmit ptDNA variants to the next generation, whereas in species with uniparental inheritance, such as oogamous brown algae, only one parent transmits variation to the next generation. If, as we propose, biparental inheritance enhances recombination of ptDNAs, this could have led to a higher level of variation by allowing variants to be combined in the same ptDNA, as we found in the simulation. This may explain why isogamous brown algae have more variable ptDNA genomes than other brown algal species.

Nonetheless, although the data presented here support the proposed link between ptDNA variation and biparental inheritance, it is important to also consider other hypothesis, such as an influence of other Ectocarpales genetic or eco-physiological features (e.g. mutation rate or effective population size). Therefore, further research needs to be directed at investigating how genomic rearrangements occur in the ptDNAs of isogamous and oogamous species to allow conclusions to be drawn about universal evolutionary trends based on the biological features of brown algal plastid genomes.

## Materials and Methods

### Sample collection and DNA extraction

Fresh thalli of *Pe. binghamiae* and *Sc. canaliculatus* were collected from an intertidal zone on the coast of Namae, Yangyang-gun, Korea (37°56′48′′N, 128°47′10′′E) on March 28, 2015. The samples were cleaned with a paper towel in the field to remove epiphytic algae and were transported to the laboratory. Due to the siphonous shape of *Sc. canaliculatus*, microalgal contaminants can be found in the internal part of the alga. Therefore, we performed two additional washes with filtered seawater of the internal part of the *Sc. canaliculatus* thalli. Cleaned samples were dried and preserved in silica gel. Voucher specimens were deposited at the Sungkyunkwan University herbarium under the accessions SSKU003989 (*Sc. canaliculatus*) and SKKU003990 (*Pe. binghamiae*), respectively.

Male and female gametophyte strains of *Ectocarpus* species were used to confirm the species-level variation and biparental inheritance of ptDNA. The male *E. siliculosus* strain (CCAP1310/131, Ec08), collected by Dieter G. Müller in Naples, Italy in 1975, is a subculture of the strain used in the experiment on artificial hybrids by Peters *et al*.^21^. A female *Ectocarpus* sp.-7 strain (Ec73), isolated in 2002 from a field sporophyte collected in San Juan de Marcona, Peru in 2002, is a sister strain of the reference genome strain Ec32^26,44^. These two *Ectocarpus* species are suitable for inter-specific analysis because they have been geographically separated recently so they are still inter-fertile. The ptDNA of strain Ec08 (male gametophyte) was sequenced in this study, as was the ptDNA of the hybrid strain Ec220, derived from a cross between Ec08 and Ec73, and the latter was compared with the parental sequences. These *Ectocarpus* strains (Ec08, Ec73, and Ec220) were cultured in Roscoff, France and sent to Korea for DNA extraction and ptDNA sequencing.

Epiphytic male and female *Sc. promiscuus* gametophytes were collected from an intertidal zone of the Botofurinai beach near the Muroran Marine Station (Field Science Center for Northern Biosphere, Hokkaido University Muroran 051–0013, Japan) on April 5, 2018. Male and female gametophytes were identified by observation of fertility under a light microscope. These two strains were selected for the intra-species-level analysis, with inter-species comparison of *Ectocarpus* species.

The *Cl. okamuranus* strain, together with nuclear sequence data, was provided by the Okinawa Prefectural Fisheries Research and Extension Center at the Okinawa Institute Science and Technology. DNA extraction was performed as described previously^45^.

All samples except *Cl. okamuranus* were frozen in liquid nitrogen and ground into a fine powder with an Automill homogenizer (Tokken, Inc., Japan). Total genomic DNA was extracted using the GeneAll plant SV mini kit (GeneAll Biotechnology, Seoul, Korea) following the manufacturer’s instructions. To remove enzyme-inhibiting compounds such as polysaccharides, a subsequent purification was performed using the PowerClean® DNA Clean-Up Kit (Qiagen, Carlsbad, CA, USA) following the manufacturer’s instructions.

### Whole genome sequencing and *de novo* assembly

Genomic DNA libraries with 400 bp inserts were constructed using the Ion Xpress Plus gDNA Fragment Library kit (Thermo Fisher Scientific, San Francisco, CA, USA). Sequencing reads were generated on an Ion Personal Genome Machine (PGM) platform with the Ion PGM Hi-Q Template kit and Ion PGM Hi-Q sequencing kit (Thermo Fisher Scientific, San Francisco, CA, USA). Raw read data were *de novo* assembled to generate contigs with the MIRA assembler v4.0.2.1, the SPAdes assembler v5.0 (embedded in the Ion Torrent Server v5.0.5) and the CLC Genomics Workbench v5.5.1 (CLC bio, Aarhus, Denmark) with default settings. Plastid and mitochondrial contigs were identified using tBLASTn with the amino acid sequences of *E. siliculosus* (GenBank accession: NC_013498.1, NC_030223.1) as a reference. Sorted contigs were assembled to construct a circular sequence using the assembly function of the Geneious R8 program^46^. To revise assembly errors, raw sequence reads were re-mapped onto the consensus sequence using CLC Genomics Workbench. Inverted repeat (IR) regions and indel errors on the consensus were corrected by PCR amplification and Sanger sequencing (Macrogen Inc., Seoul, Korea). Protein coding genes were identified using a BLASTx search against the stramenopile protein sequence database at NCBI. The RNAmmer 1.2 server^47^ and ARAGORN^48^ were used to predict rRNA and tRNA encoding sequences. All annotations were checked manually. Finalized genome maps were created with OGdraw v1.2^49^. The methods used to construct organellar genomes using NGS-derived data generally followed those used by Song *et al*.^50^.

The *Cl. okamuranus* scaffold corresponding to the ptDNA (ID: Cok_S_s60) was recovered in this study from the assembly published by Nishitsuji *et al*.^45^. The gap regions within this scaffold were sequenced using PCR amplification and Sanger sequencing (ABI3130xl), as described previously^51^. Comparison of the *Cl. okamuranus* scaffold with the plastid genome of *E. siliculosus* using a LASTZ alignment^52^ (plugin tool of Geneious R8 program) showed that the scaffold was chimeric with partial plastid and mitochondrial genome sequences. The partial contigs of ptDNA and mitochondrial genome (mtDNA) were restored and circularized using bioinformatics tools described previously.

### Phylogenomics and co-lineararity analysis

A core set of 137 plastid protein coding genes from 15 species (a list of these genes can be found in Table S2) and 36 mitochondrial genes from 35 species were used for phylogenomic analysis. Translated protein sequences of genes were aligned using MAFFT version 7.110^53^, then concatenated into one dataset. A maximum likelihood tree was constructed using IQ-Tree v1.3.0^54^. An independent substitution model was assigned to each partition (i.e., gene) in the dataset using the IQ-Tree built-in model test option (-m TEST). Branch supports were calculated with the ultrafast bootstrap approximation (-bb 1,000) implemented in IQ-Tree.

The total amount of the non-coding sequence in the entire ptDNA sequence was calculated manually, by summing the lengths of all protein-coding sequences (CDSs) and subtracting this value from the full ptDNA length. To compare genomic structures, the complete sequence of each organellar genome was aligned with the progressiveMauve algorithm of Mauve v2.3.1^55^ with default settings. The output backbone file was visualized, together with the generated treefile, using the R library genoPlotR^56^.

For the comparison of the two *Ectocarpus* species, nucleotide substitution frequency was measured with a 100 bp window and plotted on the full-length sequence. Nucleotide insertions and deletions were treated as substitution events. To confirm biparental inheritance of ptDNA, we sequenced a hybrid that had been derived by crossing individuals from two *Ectocarpus* species and mapped the reads onto each respective parental genome.

### Simulation of plastid inheritance in two virtual populations

To model ptDNA variation over many generations, we set two virtual populations consisting of individuals that with biparentally-inherited plastids (BiP population) and uniparentally-inherited plastids (UniP population), respectively. In each generation, one plastid was selected from each of the maternal and paternal individuals (in the BiP population) or from only the maternal individual (in the UniP population) during the meiosis step. In the biparental population, the probability of selecting a plastid to be inherited was chosen randomly among one of these: 0.25, 0.5, and 0.75, because plastids distribute irregularly in the four meiotic daughter cells^21^ and the probability that a gamete contains a particular ptDNA may be determined by the initial proportion. We assumed that the proportions of each ptDNA would be maintained in the individuals of the BiP population in the next generation. Other variables such as mortality, population size growth, and the mutation rate of ptDNA were applied equally to the two populations. We assumed that one in every 100 plastid genomes was modified by a mutation (0.01 probability). Among the gametes, two (in the BiP population) or one (in the UniP population) transmits its ptDNA to the next generation. Finally, the population size was reduced (with 0.5 mean, normally distributed with 0.01 standard deviation of mortality) after each generation to assume a saturated population. It was also assumed that there was no benefit for survival either in BiP and UniP populations, and so no selection pressure from natural selection. The number of differentiated ptDNA molecules, i.e. ptDNA molecules that differ from the original sequence as a result of the introduction of mutations at a given rate, was measured as ptDNA diversity. Under the assumption of a doubling of population size every generation before the effect of mortality (more or less than 0.5), total population size generally increased with each generation. Figure 5B shows the result, obtained during 200 generations, of the simulation we executed with 200 initial individuals and replicated 100 times.

## Supplementary information

Supplementary information.

Supplementary information 2.

Supplementary information 3.

## Data Availability

Newly sequenced plastid genomes (ptDNAs) of *Cl. okamuranus* (MG739403), *Pe. binghamiae* (MG460360), *Sc. canaliculatus* (MF591718) and *Sc. promiscuus* (female: MK107984, male: MK107985), *E. siliculosus* (MN181444) and mitochondrial genomes (mtDNAs) of *Cl. okamuranus* (MG488292) and *Pe. binghamiae* (MG488291) have been deposited in the NCBI GenBank database.
