# Supplementary material for: Organelle inheritance and genome architecture variation in isogamous brown algae
Source: Sci Rep. 2020 Feb 6;10:2048. doi: 10.1038/s41598-020-58817-7 (PMC7005149; doi:10.1038/s41598-020-58817-7)
Supplement: Supplementary file 1 — Supplementary information. [file 41598_2020_58817_MOESM1_ESM.pdf]

Supplementary Information

Organelle inheritance and genome architecture variation in isogamous and anisogamous brown algae

Ji Won Choi[1], Louis Graf[1], Akira F. Peters[2], J. Mark Cock[3], Koki Nishitsuji[4], Asuka Arimoto[4], Eiichi Shoguchi[4], Chikako Nagasato[5], Chang Geun Choi[6], and Hwan Su Yoon[1]*

[1]Department of Biological Sciences, Sungkyunkwan University, Suwon 16419, Korea

[2]Bezhin Rosko, 29250 Santec, France

[3]Algal Genetics Group, UMR 8227, CNRS, Sorbonne Universités, UPMC, Station Biologique Roscoff, CS 90074, 29688 Roscoff, France

[4]Marine Genomics Unit, Okinawa Institute of Science and Technology Graduate University, Onna, Okinawa 904-0495, Japan

[5]Muroran Marine Station, Field Science Center for Northern Biosphere, Hokkaido University Muroran 051-0013, Japan

[6]Department of Ecological Engineering, Pukyong National University, Busan 48513, Korea

*Corresponding author

E-mail: hsyoon2011@skku.edu

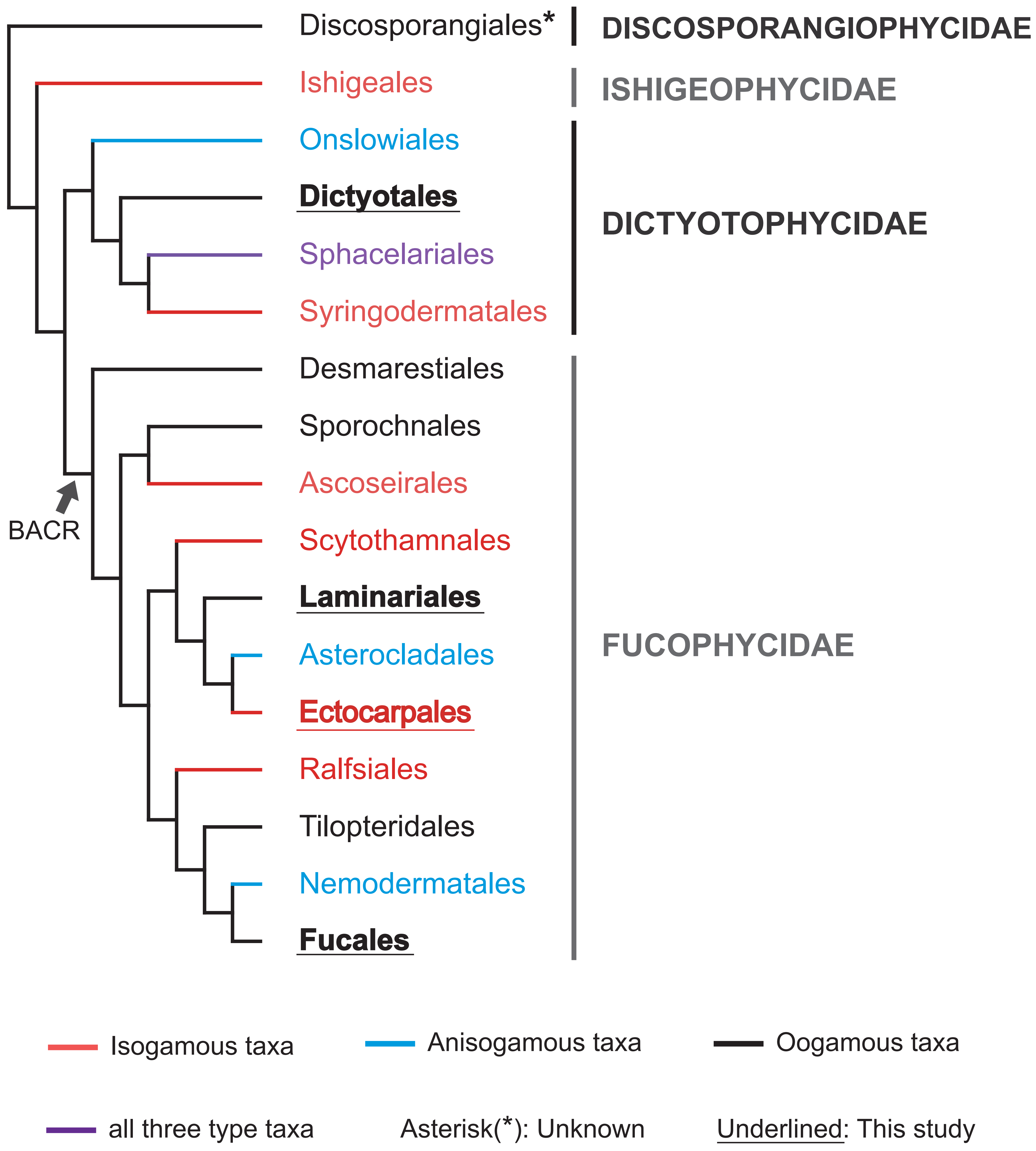

Fig. S1. Classification of four subclasses of Phaeophyceae and the type of fertilization at the order level. (BACR: Brown algal crown radiation)

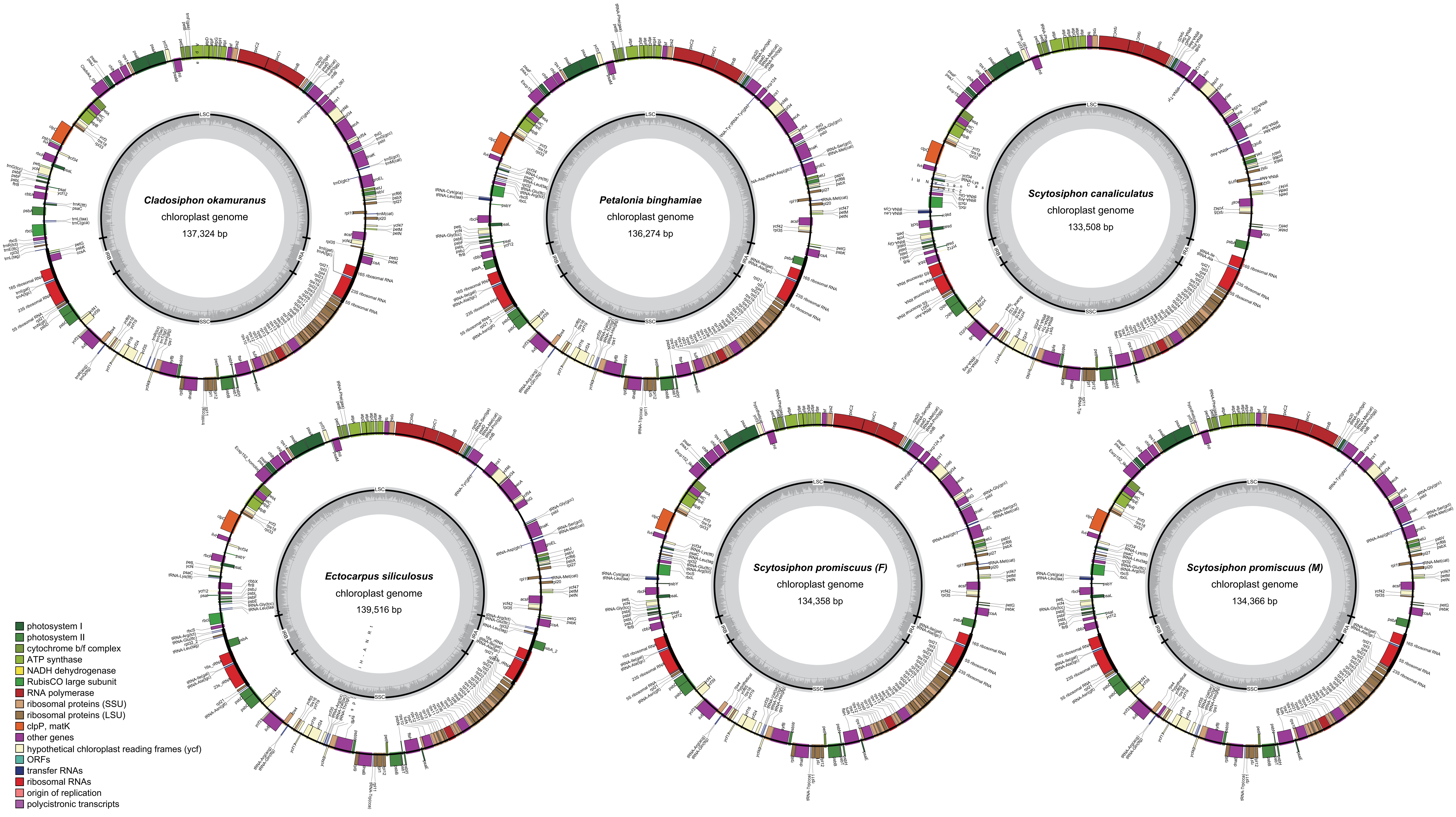

Fig. S2. Circular maps of the plastid genomes of four Ectocarpales species.

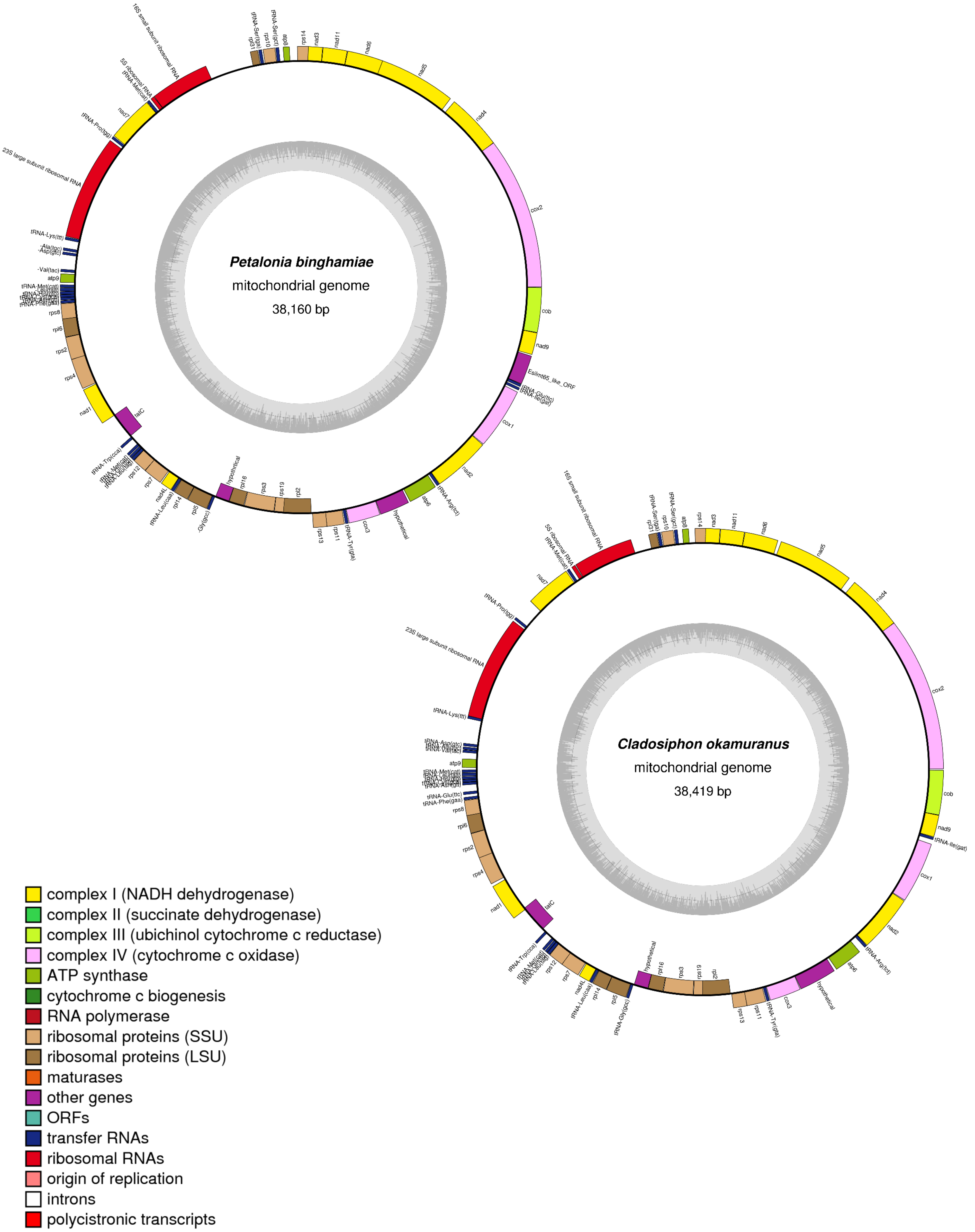

Fig. S3. Circular maps of the mitochondrial genomes of two Ectocarpales species.

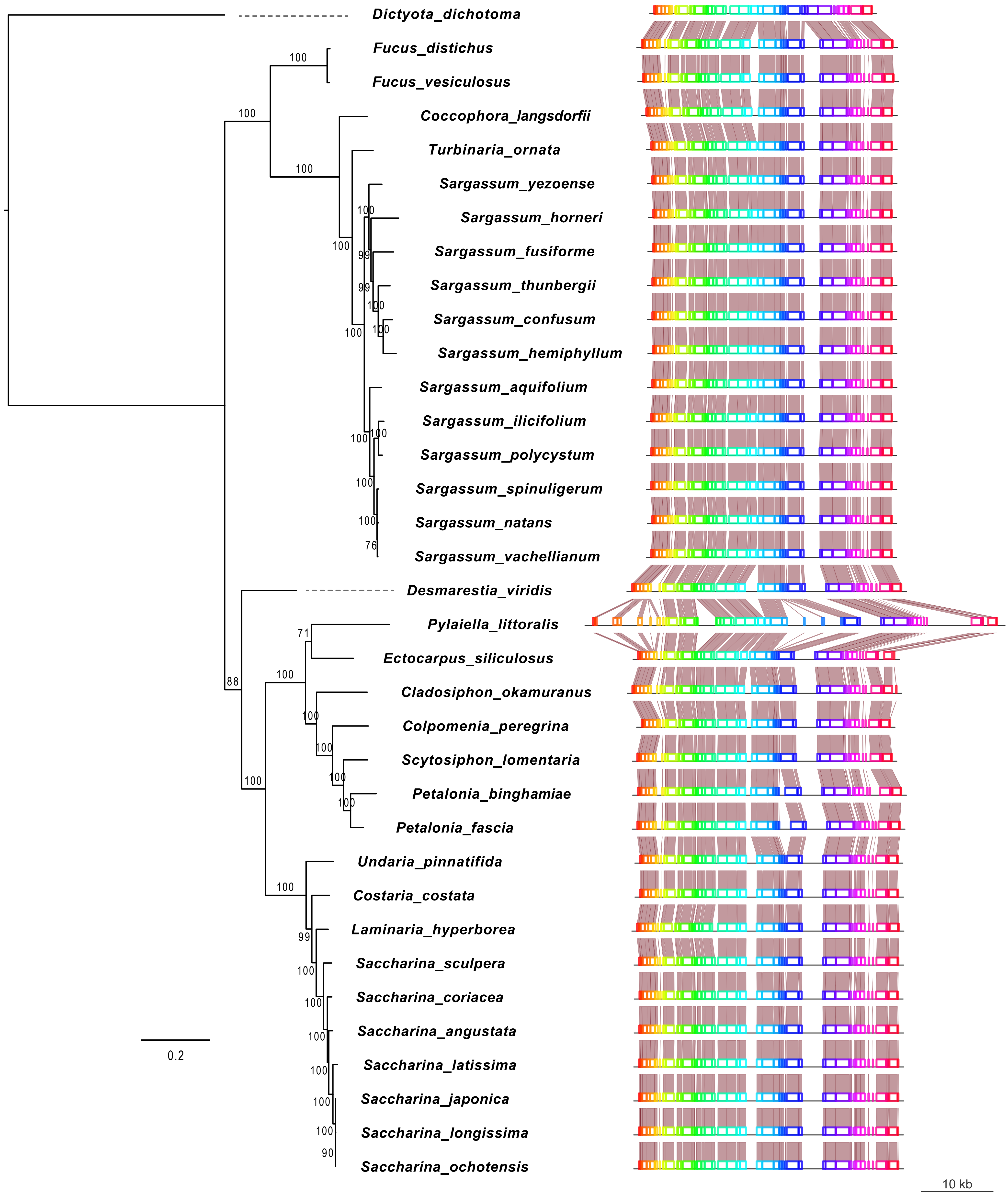

Fig. S4. Co-linear alignment and phylogeny-based clustering of mtDNAs of 32 brown algal species

Table. S1. Plastid genome sequncing statistics

	Scytosiphon canaliculatus	***Scytosiphon promiscuus*** **(♂)**	***Scytosiphon promiscuus*** **(♀)**	***Petalonia binghamiae***	***Ectocarpus siliculosus*** **(♂)**	***Cladosiphon okamuranus***[1]
NGS Platform	Ion PGM	Ion PGM	Ion PGM	Ion PGM	Ion PGM	Illumina
No. reads	4,066,433	4,511,276	7,604,996	4,232,288	7,659,092	46,686,028
Total length (Mb)	1,137	1,375	2,781	1,028	2,752	13,916
N50 contig (bp)[2]	2,478	4,860	1,829	3,533	8,679	21,705
Average coverage	193.5	371.2	494.8	470.9	159.9	99.4
Reference	This study	This study	This study	This study	This study	Nishitsuji et al. and this study

[1]The whole genome sequencing of *Cladosiphon okamuranus* was conducted in Nishitsuji et al. (2016).

[2]The N50 value was calculated from the merged all assemblies

Table S2. Protein coding gene contents of plastid genomes used in the phylogenomic analysis. Presence of genes are indicated as filled boxes in each species (Duplicated genes are filled darker). Genes are categorized by their functions. Unidentified open reading frames (orf) are excluded. A 137 core genes are written in red.

Functional Categories	Protein Coding Gene	Count	Phaeophyceae													
			Fucales				Laminariales			Ectocarpales						Dictyotales
			Coccophora.langsdorfii	*Sargassum.horneri*	*Sargassum.thunbergii*	*Fucus.vesiculosus*	*Costaria.costata*	*Saccharina.japonica*	*Undaria.pinnatifida*	*Pleurocladia.lacustris*	*Ectocarpus.siliculosus*	*Cladosiphon.okamuranus*	*Scytosiphon.canaliculatus*	*Scytosiphon.lomentaria*	*Petalonia.binghamiae*	*Dictyopteris.divaricata*
Transcription	cbbX	14	+	+	+	+	+	+	+	+	+	+	+	+	+	+
	rbcR	13	+	+	+	+	+	+	+	+	+	+	+	+	+	
	rpoA	14	+	+	+	+	+	+	+	+	+	+	+	+	+	+
	rpoB	14	+	+	+	+	+	+	+	+	+	+	+	+	+	+
	rpoC1	14	+	+	+	+	+	+	+	+	+	+	+	+	+	+
	rpoC2	14	+	+	+	+	+	+	+	+	+	+	+	+	+	+
	tsf	14	+	+	+	+	+	+	+	+	+	+	+	+	+	+
Translation	tufA	14	+	+	+	+	+	+	+	+	+	+	+	+	+	+
	syfB	10					+	+	+	+	+	+	+	+	+	+
Ribosomal proteins large subunit	rpl1	14	+	+	+	+	+	+	+	+	+	+	+	+	+	+
	rpl2	14	+	+	+	+	+	+	+	+	+	+	+	+	+	+
	rpl3	14	+	+	+	+	+	+	+	+	+	+	+	+	+	+
	rpl4	14	+	+	+	+	+	+	+	+	+	+	+	+	+	+
	rpl5	14	+	+	+	+	+	+	+	+	+	+	+	+	+	+
	rpl6	14	+	+	+	+	+	+	+	+	+	+	+	+	+	+
	rpl9	14	+	+	+	+	+	+	+	+	+	+	+	+	+	+
	rpl11	14	+	+	+	+	+	+	+	+	+	+	+	+	+	+
	rpl12	14	+	+	+	+	+	+	+	+	+	+	+	+	+	+
	rpl13	14	+	+	+	+	+	+	+	+	+	+	+	+	+	+
	rpl14	14	+	+	+	+	+	+	+	+	+	+	+	+	+	+
	rpl16	14	+	+	+	+	+	+	+	+	+	+	+	+	+	+
	rpl18	14	+	+	+	+	+	+	+	+	+	+	+	+	+	+
	rpl19	14	+	+	+	+	+	+	+	+	+	+	+	+	+	+
	rpl20	14	+	+	+	+	+	+	+	+	+	+	+	+	+	+
	rpl21	14	+	+	+	+	+	+	+	++	++	++	++	++	++	++
	rpl22	14	+	+	+	+	+	+	+	+	+	+	+	+	+	+
	rpl23	14	+	+	+	+	+	+	+	+	+	+	+	+	+	+
	rpl24	14	+	+	+	+	+	+	+	+	+	+	+	+	+	+
	rpl27	14	+	+	+	+	+	+	+	+	+	+	+	+	+	+
	rpl29	14	+	+	+	+	+	+	+	+	+	+	+	+	+	+
	rpl31	14	+	+	+	+	+	+	+	+	+	+	+	+	+	+
	rpl32	12	+	+	+	+	+	+	+		++	+	+	+	+	
	rpl33	14	+	+	+	+	+	+	+	+	+	+	+	+	+	+
	rpl34	14	+	+	+	+	+	+	+	+	+	+	+	+	+	+
	rpl35	14	+	+	+	+	+	+	+	+	+	+	+	+	+	+
	rpl36	14	+	+	+	+	+	+	+	+	+	+	+	+	+	+
Ribosomal proteins small subunit	rps1	14	+	+	+	+	+	+	+	+	+	+	+	+	+	+
	rps2	14	+	+	+	+	+	+	+	+	+	+	+	+	+	+
	rps3	14	+	+	+	+	+	+	+	+	+	+	+	+	+	+
	rps4	14	+	+	+	+	+	+	+	+	+	+	+	+	+	+
	rps5	14	+	+	+	+	+	+	+	+	+	+	+	+	+	+
	rps7	14	+	+	+	+	+	+	+	+	+	+	+	+	+	+
	rps8	14	+	+	+	+	+	+	+	+	+	+	+	+	+	+
	rps9	14	+	+	+	+	+	+	+	+	+	+	+	+	+	+
	rps10	14	+	+	+	+	+	+	+	+	+	+	+	+	+	+
	rps11	14	+	+	+	+	+	+	+	+	+	+	+	+	+	+
	rps12	14	+	+	+	+	+	+	+	+	+	+	+	+	+	+
	rps13	14	+	+	+	+	+	+	+	+	+	+	+	+	+	+
	rps14	14	+	+	+	+	+	+	+	+	+	+	+	+	+	+
	rps16	14	+	+	+	+	+	+	+	+	+	+	+	+	+	+
	rps17	14	+	+	+	+	+	+	+	+	+	+	+	+	+	+
	rps18	14	+	+	+	+	+	+	+	+	+	+	+	+	+	+
	rps19	14	+	+	+	+	+	+	+	+	+	+	+	+	+	+
	rps20	14	+	+	+	+	+	+	+	+	+	+	+	+	+	+
Energy Metabolism-ATP synthase	atpA	14	+	+	+	+	+	+	+	+	+	+	+	+	+	+
	atpB	14	+	+	+	+	+	+	+	+	+	+	+	+	+	+
	atpD	14	+	+	+	+	+	+	+	+	+	+	+	+	+	+
	atpE	14	+	+	+	+	+	+	+	+	+	+	+	+	+	+
	atpF	14	+	+	+	+	+	+	+	+	+	+	+	+	+	+
	atpG	14	+	+	+	+	+	+	+	+	+	+	+	+	+	+
	atpH	14	+	+	+	+	+	+	+	+	+	+	+	+	+	+
	atpI	14	+	+	+	+	+	+	+	+	+	+	+	+	+	+
Photosynthesis-Photosystem I	psaA	14	+	+	+	+	+	+	+	+	+	+	+	+	+	+
	psaB	14	+	+	+	+	+	+	+	+	+	+	+	+	+	+
	psaC	14	+	+	+	+	+	+	+	+	+	+	+	+	+	+
	psaD	14	+	+	+	+	+	+	+	+	+	+	+	+	+	+
	psaE	14	+	+	+	+	+	+	+	+	+	+	+	+	+	+
	psaF	14	+	+	+	+	+	+	+	+	+	+	+	+	+	+
	psaI	14	+	+	+	+	+	+	+	+	+	+	+	+	+	+
	psaJ	14	+	+	+	+	+	+	+	+	+	+	+	+	+	+
	psaL	14	+	+	+	+	+	+	+	+	+	+	+	+	+	+
	psaM	14	+	+	+	+	+	+	+	+	+	+	+	+	+	+

Table S2. continued

Functional Categories	Protein Coding Gene	Count	Phaeophyceae													
			Fucales				Laminariales			Ectocarpales						Dictyotales
			Coccophora.langsdorfii	*Sargassum.horneri*	*Sargassum.thunbergii*	*Fucus.vesiculosus*	*Costaria.costata*	*Saccharina.japonica*	*Undaria.pinnatifida*	*Pleurocladia.lacustris*	*Ectocarpus.siliculosus*	*Cladosiphon.okamuranus*	*Scytosiphon.canaliculatus*	*Scytosiphon.lomentaria*	*Petalonia.binghamiae*	*Dictyopteris.divaricata*
Photosystem II	psbA	14														
	psbB	14														
	psbC	14														
	psbD	14														
	psbE	14														
	psbF	14														
	psbH	14														
	psbI	14														
	psbJ	13														
	psbK	14														
	psbL	14														
	psbN	14														
	psbT	14														
	psbV	14														
	psbW	14														
	psbX	14														
	psbY	14														
Cytochrome complex	petA	14														
	petB	14														
	petD	14														
	petF	14														
	petG	14														
	petJ	14														
	petL	11														
	petM	14														
	petN	14														
	ccs1	14														
	ccsA	14														
Metabolism	chlI	14														
	chlB	14														
	chlL	14														
	chlN	14														
	rbcL	14														
	rbcS	14														
	thiG	14														
	thiS (ycf40)	14														
Cellular process	clpC	14														
	dnaB	14														
	dnaK	14														
	ftsH	14														
	groEL	14														
	secA	14														
	secG (ycf47)	14														
	secY	14														
	sufB (ycf24)	14														
	sufC (ycf16)	14														
	tatC	14														
	acsF	14														
	ftrB	14														
	ilvB	14														
	ilvH	14														
Conserved proteins	ycf3	14														
	ycf4	14														
	ycf12	14														
	ycf17	10														
	ycf19	14														
	ycf33	14														
	ycf34	14														
	ycf35	14														
	ycf37	14														
	ycf39	14														
	ycf41	14														
	ycf42	14														
	ycf46	14														
	ycf54	10														
	ycf65	14														
	ycf66	14														
	Escp124_like	14														
	Escp152_like	14														
Total	72		139	139	139	139	139	139	139	141	144	144	143	143	144	139
